# microRNA-9-5p protects liver sinusoidal endothelial cell against oxygen glucose deprivation/reperfusion injury

**DOI:** 10.1515/biol-2021-0042

**Published:** 2021-04-19

**Authors:** Yi Duan, Yuanyuan Meng, Zhifeng Gao, Xiaoyu Wang, Huan Zhang

**Affiliations:** Department of Anesthesiology, Beijing Tsinghua Changgung Hospital, School of Clinical Medicine, Tsinghua University, No. 168 Litang Road, Beijing 102218, China

**Keywords:** microRNA-9-5p, CXC chemokine receptor-4, sinusoidal endothelial cell, ischemia-reperfusion injury, OGD/HG injury

## Abstract

**Background:**

Maintenance of the function and survival of liver sinusoidal endothelial cells (LSECs) play a crucial role in hepatic ischemia/reperfusion (I/R) injury, a major cause of liver impairment during the surgical treatment. Emerging evidence indicates a critical role of microRNAs in I/R injury. This study aims to investigate whether miR-9-5p exerts a protective effect on LSECs.

**Methods:**

We transfected LSECs with miR-9-5p mimic or mimic NC. LSECs were treated with oxygen and glucose deprivation (OGD, 5% CO_2_, and 95% N_2_), followed by glucose-free Dulbecco’s modified Eagle’s medium (DMEM) medium for 6 h and high glucose (HG, 30 mmol/L glucose) DMEM medium for 12 h. The biological role of miR-9-5p in I/R-induced LSEC injury was determined.

**Results:**

In the *in vitro* model of OGD/HG injury in LSECs, the expression levels of miR-9-5p were significantly downregulated, and those of CXC chemokine receptor-4 (CXCR4) upregulated. LSEC I/R injury led to deteriorated cell death, enhanced oxidative stress, and excessive inflammatory response. Mechanistically, we showed that miR-9-5p overexpression significantly downregulated both mRNA and protein levels of CXCR4, followed by the rescue of LSECs, ameliorated inflammatory response, and deactivation of pro-apoptotic signaling pathways.

**Conclusions:**

miR-9-5p promotes LSEC survival and inhibits apoptosis and inflammatory response in LSECs following OGD/HG injury via downregulation of CXCR4.

## 1 Introduction

Hepatic ischemia/reperfusion (I/R) injury, a major complication of liver surgeries, liver transplantation, and hemorrhagic shock, is attributable to deteriorated post-operational hepatic dysfunction, extended hospital stay, as well as aggravated morbidity and mortality [1,2,3]. A crucial event during this process is the death of liver sinusoidal endothelial cells (LSECs), which takes place only a few minutes following reperfusion, several hours before detectable hepatocyte death [1,4]. Apoptosis and inflammatory responses are pivotal underlying mechanisms of I/R-induced endothelial damage [5,6,7]. LSECs, which line the primary barrier between the hepatocytes and the bloodstream, play complex roles and are prone to injury [8]. Therefore, understanding the biological function of LSECs is essential to elucidate liver pathology during I/R injury.

One potential mechanism of LSEC survival is the alterations of epigenetic regulators, particularly microRNAs (miRNAs) [9]. miRNAs usually regulate target functional proteins via binding to complementary seed sequences in 3′ untranslated regions (3′-UTRs) of mRNA, leading to their degradation or inhibition of translation [10,11,12]. miR-9-5p has been extensively studied in malignant diseases, such as oral squamous cell carcinoma, nasopharyngeal carcinoma, papillary thyroid cancer, and prostate cancer [13,14,15,16,17]. Depending on the specific cell type and pathological conditions, miR-9-5p may exert either pro-apoptotic or anti-apoptotic functions. We have previously shown that miR-9-5p protected human umbilical vascular endothelial cells (HUVECs) against apoptosis and excessive inflammatory response via downregulation of CXC chemokine receptor-4 (CXCR4) [18]. Nevertheless, the precise biological role of miR-9-5p in LSECs remains unknown.

We herein hypothesized that miR-9-5p might play a critical role in the attenuation of oxygen and glucose deprivation/high glucose (OGD/HG)-induced LSEC impairment *in vitro*. In this study, we have established an *in vitro* model by incubating LSECs with OGD followed by HG medium to mimic reperfusion. This enables us to observe the intracellular effects of OGD/HG injury in LSCEs. We then tested whether overexpression of miR-9-5p sufficiently ameliorates activated apoptosis, inflammatory response, and cell death in OGD/HG-treated LSECs.

## 2 Materials and methods

### 2.1 *In vitro* model of I/R and cell transfection

Briefly, LSECs (Ningbo Mingzhou Biotechnology Co. Ltd, MZ-M0395) were incubated in an Eppendorf Galaxy 170 R incubator under hypoxia conditions (5% CO_2_ and 95% N_2_ at 5 L/min) for 30 min till oxygen concentration reached 1%. The hypoxia mixture gas was continuously injected to maintain O_2_ concentration in the incubator below 1%. Then, cells were treated with glucose-free Dulbecco’s modified Eagle’s medium (DMEM) for 6 h, followed by HG (30 mmol/L glucose) DMEM medium containing 10% fetal bovine serum (FBS) at 5% CO_2_ for 12 h. The sequences of miR-9-5p mimic and miR-9-5p mimic NC were provided by the Shanghai GenePharma Co., Ltd (Shanghai, China). LSECs were inoculated into a 6-well plate 24 h before transfection. Upon reaching approximately 50% of cell confluence, the cells were incubated with 200 μL of transfection reagent and plasmids, as per the instructions in the manual from Lipofectamine 2000 transfection kit (11668-027; Invitrogen Inc., Carlsbad, CA, USA). The medium was changed after 6 h and collected after 48 h, according to the manufacturer’s instructions.

### 2.2 RNA isolation and quantitation

Total RNA of transfected cells was isolated using the Trizol reagent according to the manufacturer’s protocol; 2 µg of RNA was reverse transcribed into cDNA. The primers of miR-9-5p and CXCR4 were designed and synthesized by Beijing Augct Biotechnology Co., Ltd (Beijing, China), with U6 acting as the internal reference for miR-9-5p, and glyceraldehyde-3-phosphate dehydrogenase (GAPDH) for CXCR4. The relative transcriptase mRNA levels of the target gene were calculated by conducting the relative quantitative method:

\text{ΔCT}=\text{Ct}\hspace{.5em}\text{target}\hspace{.5em}\text{gene}-\text{Ct}\hspace{.5em}\text{internal}\hspace{.5em}\text{reference}\text{.}]

### 2.3 Western blot analysis

Proteins were extracted from cultured LSECs and homogenized in lysis buffer. Following lysate centrifugation at 12,000 rpm for 10 min, the supernatant was collected, and the concentration of protein was determined using a protein quantitative kit, adjusting protein concentration to the same level. Proteins were treated with the sample buffer and prepared on 15% separation gel. After 2 h of protein electrophoresis, proteins were transferred to the polyvinylidene fluoride (PVDF) membranes. The membranes were blocked with 5% of skimmed milk powder for 2 h at room temperature on a horizontal shaking table. Then, the membranes were incubated with primary antibodies: CXCR4 (1:100, ab124824), Bcl-2-associated X protein (Bax) (1:1,000, ab32503), B-cell lymphoma 2 (Bcl-2) (1:2,000, ab182858), cleaved caspase 3 (1:500, ab32042), cleaved caspase 9 (1:1,000, ab2324), and β-actin (1:2,000, ab8227) overnight at 4°C. All antibodies were purchased from Abcam Inc. (Cambridge, MA, USA). PVDF membranes were then washed using a phosphate buffer saline Tween-20 (PBST; phosphate buffer saline [PBS] containing 0.1% Tween-20) four times for 15 min, followed by incubation with a secondary antibody horseradish peroxidase (HRP)-labeled goat anti-rabbit IgG antibody (1:3,000) for 1 h at room temperature. Membranes were treated with enhanced chemiluminescence (ECL) agent before photographed and analyzed using Jetta image analysis system. The relative protein expression was defined as the ratio of the gray value of the target protein band to the internal reference β-actin or GAPDH.

### 2.4 Detection of malondialdehyde (MDA) content

The MDA content was detected by the thiobarbituric acid method following the instructions provided by the MDA kit. Cells were inoculated in a 24-well plate (four wells setup for each experimental group) with the density rate of approximately 1 × 10^5^ cells per well, followed by grouping and processing using methods mentioned above. From the cultured cells, 100 μL supernatant of each group was collected. The optical density (OD) was measured at 532 nm wavelength by a 752-type ultraviolet grating spectrophotometer. The MDA content of each group was calculated.

### 2.5 Detection of glutathione peroxidase (GSH-PX) activity

GSH-PX activity was evaluated using a GSH-PX kit based on the 5-5′-dithio-2-dinitrobenzoicacid (DTNB) method, according to the manufacturer’s instructions. Cells were inoculated into 24-well plates (four wells in each experimental group 1 × 10^5^ cells per well). Of each group, 100 μL supernatant was collected, and the absorbance value was measured at 412 nm wavelength. Three independent experiments were performed.

### 2.6 Cell counting kit-8 (CCK-8) assay

When the cell confluency reached approximately 80%, the cells were treated with 0.25% trypsin to prepare the single-cell suspension. After counting, the cells were inoculated in a 96-well plate (200 μL/well) with the density amounting to 3 × 10^3^ to 6 × 10^3^ cells/well, and six replicates were established. Cells were cultured in an incubator, and the cell proliferation was exanimated at 0, 24, 48, and 72 h. To the cells in each well 10 μL CCK-8 was added, followed by culturing for 2 h. The OD values were measured at 450 nm wavelength by the enzyme-linked immunosorbent assay (ELISA) reader. Cell viability curve was drawn (time point as the abscissa, OD value as the ordinate). The proliferative ability of cells was determined.

### 2.7 ELISA

A commercially available ELISA kit was used to test for the expression levels of interleukin-1 (IL-1), IL-6, and 3w?>tumor necrosis factor-α (TNF-α), following the manufacturer’s protocol. We measured OD values at 450 nm wavelength using a Swiss TECAN multifunctional ELISA reader.

### 2.8 Statistical analysis

The statistical analysis was conducted using the SPSS 21.0 software (IBM Corp. Armonk, NY, USA). Data were tested conforming to normal distribution and homogeneity of variance. The measurements were presented by the mean ± standard deviation. Comparisons made between two groups were performed using *t*-test, whereas comparisons among multiple groups were analyzed using one-way analysis of variance (ANOVA). Pairwise comparisons in multiple groups were performed using Tukey post-test data. The repeated measure ANOVA was used for the analysis of the data at different time points. All the experiments were performed thrice. *P* < 0.05 was considered statistically different.

## 3 Results

### 3.1 miR-9-5p is downregulated and CXCR4 upregulated in an I/R injury model *in vitro*

**Figure 1:**
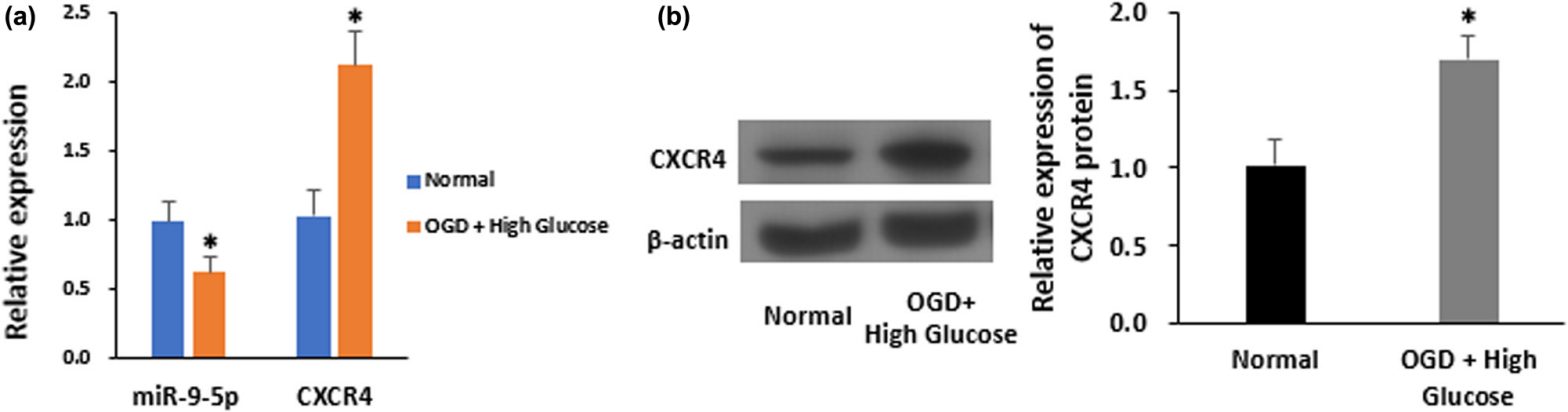
OGD/HG damage alters expression levels of miR-9-5p and CXCR4 in cultured LSECs. (a) Expression levels of miR-9-5p and CXCR4 in LSECs after OGD followed by 12 h of HG treatment, as determined by quantitative RT-qPCR. (b) Immunoblotting of CXCR4 in OGD/HG-treated LSECs (left) and quantification of CXCR4 levels (right, *n* = 3). *P < 0.05 versus control. The data represent the mean ± standard deviation.

We generated an experimental model of I/R injury *in vitro* by incubating LSECs with OGD (5% CO_2_ and 95% N_2_), which imitates the low oxygen and glucose conditions during pathological conditions such as liver surgery. The LSECs were then treated with glucose-free DMEM medium for 6 h, followed by HG (30 mmol/L glucose) DMEM medium for 12 h. RT-qPCR and western blot analysis were used to detect the expression levels of miR-9-5p and CXCR4 in LSECs, respectively. Intriguingly, I/R suppressed the levels of miR-9-5p by 37.4% but enhanced that of CXCR4 by 2.05-fold *in vitro* (Figure 1a). This expression pattern was further confirmed by the detection of CXCR4 protein expression levels post-I/R injury in LSECs (Figure 1b). Thus, these data indicate that miR-9-5p and CXCR4 may exert functional roles in I/R injury in LSECs. Their precise biological impacts, however, remain to be explored.

### 3.2 OGD/HG induces LSEC injury

**Figure 2:**
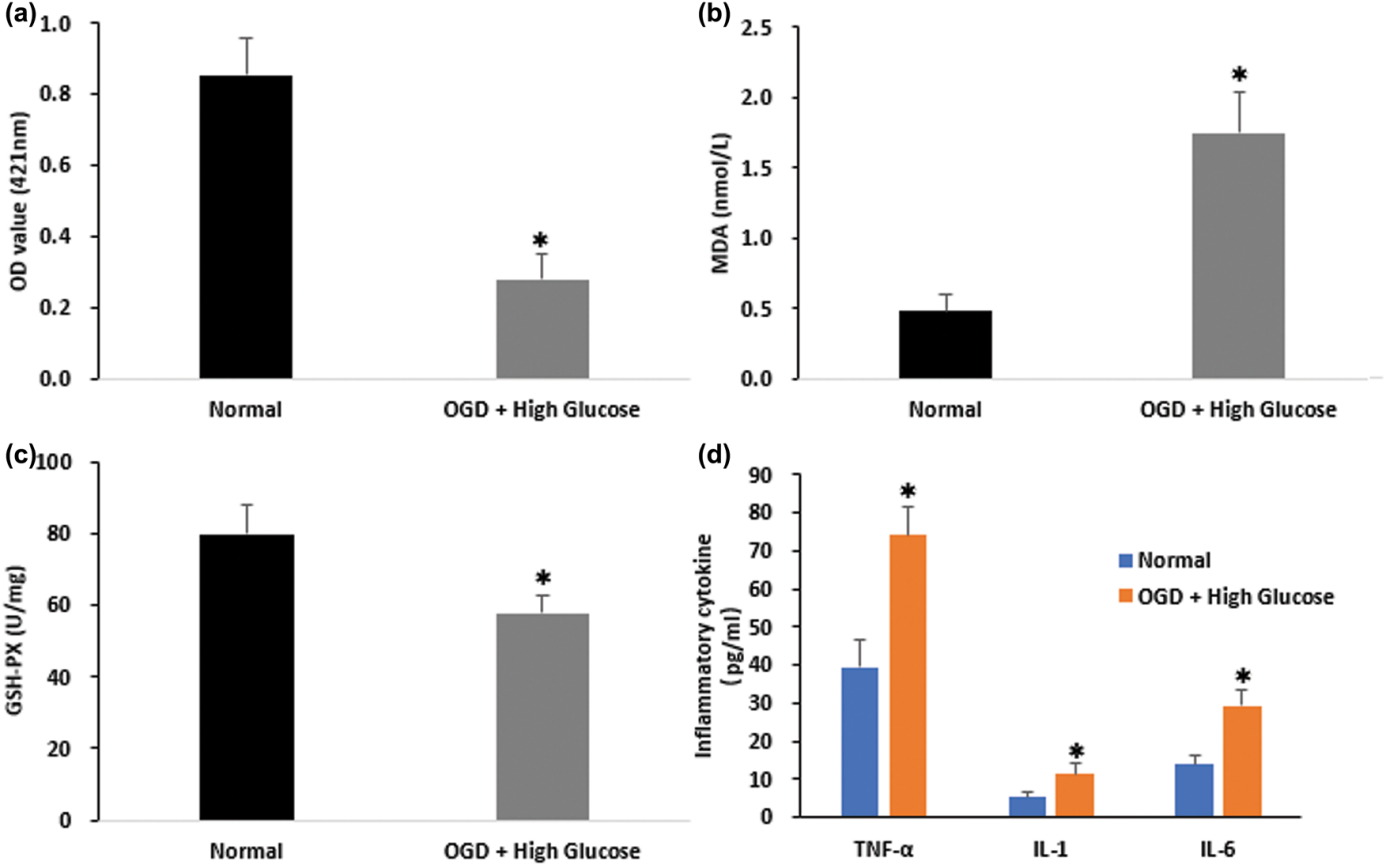
OGD/HG damage deteriorates cell survival and promotes oxidative stress and inflammatory response in LSECs. (a) Cell viability assay was performed on LSCEs subjected to OGD/HG insults. (b) MDA contents were detected in LSCEs following OGD/HG damage. (c) GSH-PX activity was tested in LSCEs following OGD/HG damage. (d) Expression levels of TNF-α, IL-1, and IL-6 in control and OGD/HG group as determined by ELISA. *P < 0.05 versus control. The data represent the mean ± standard deviation.

Next, we sought to evaluate the effects of OGD/HG insults on LSECs. Cell viability was determined using a non-radioactive CCK-8 assay. Accordingly, OGD/HG injury significantly reduced LSEC survival by 67.3% (Figure 2a). Given that oxidative stress, inflammatory response, and apoptosis are essential pathological processes that underlie liver I/R injury, we investigated these mechanisms in our LSEC model *in vitro*. The oxidative degradation of lipids, detected by the OD value of MDA, results in free radicals taking electrons from the lipids, leading to cell damage. Thus, it is essential to assess oxidative stress. The GSH-PX plays a crucial role in the protection of liver tissues from oxidative damage, and their activities were also examined and quantified. We observed a significant elevation of OD value of MDA (3.62-fold; Figure 2b) and an ∼27.5% decrease in GSH-PX content (Figure 2c). Indeed, these data demonstrate that OGD/HG injury in LSECs led to enhanced oxidative stress. Then, we examined the impact of this injury on inflammatory response in LSECs. Using ELISA analyses, we detected expression levels of crucial pro-inflammatory factor, namely TNF-α, IL-1 and IL-6. As expected, their expression levels were all significantly increased (Figure 2d). Collectively, OGD/HG insults in LSECs aggravated cell oxidative stress and inflammatory response, leading to worsened cell survival rate.

### 3.3 Overexpression of miR-9-5p largely mitigated OGD/HG LSEC injury

**Figure 3:**
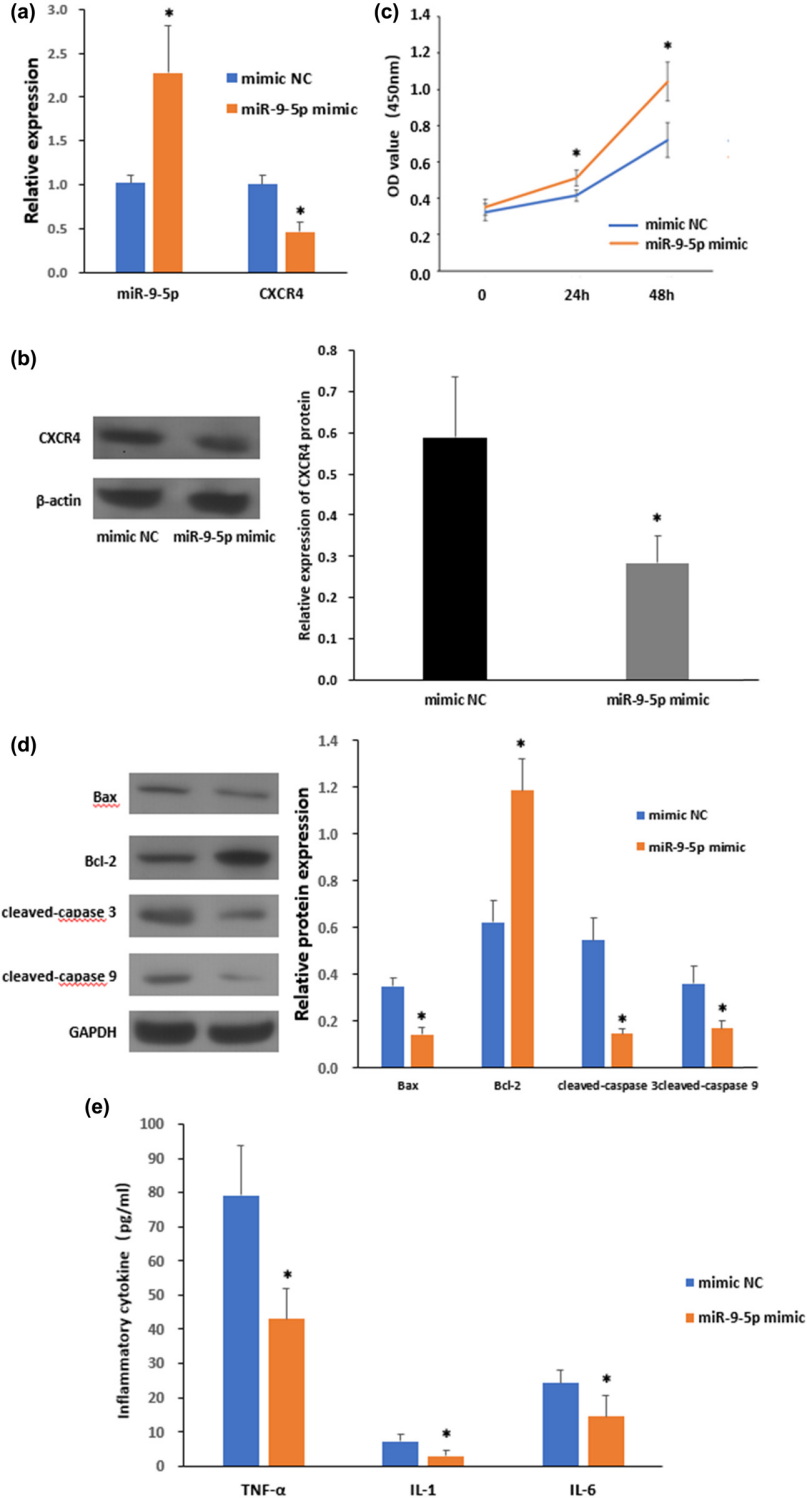
Overexpression of miR-9-5p ameliorates OGD/HG-induced LSECs impairment. (a) Expression levels of miR-9-5p and CXCR4 in LSECs following mimic NC or miR-9-5p mimic transfection, as determined by quantitative RT-qPCR. (b) Immunoblotting of CXCR4 in I/R-treated LSECs (left) and quantification of CXCR4 levels (right, *n* = 3). (c) Cell viability assay was performed on LSCEs at indicated time points following OGD/HG insults. (d) Immunoblotting of Bax, Bcl-2, cleaved-caspase 3, and cleaved-caspase 9 in OGD/HG-treated LSECs (right) and quantification of CXCR4 levels (left, *n* = 3). (e) Expression levels of TNF-α, IL-1, and IL-6 in control and OGD/HG group as determined by ELISA. *P < 0.05 versus mimic NC. The data represent the mean ± standard deviation.

**Figure 4:**
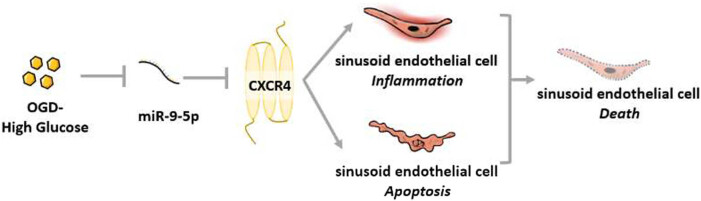
Proposed mechanism of miR9-5p-mediated protection in LSEC following OGD/HG challenge.

To investigate the functional role of miR-9-5p in OGD/HG-induced LSEC injury, we further tested these effects in the setting of miR-9-5p overexpression. LSECs were transfected with empty mimic NC or miR-9-5p mimic (Figure 3a). RT-qPCR confirmed the efficacy of transfection, as indicated by a 2.3-fold increase in miR-9-5p expression levels. Importantly, miR-9-5p overexpression significantly reduced the mRNA expression levels of CXCR4 to 53.9% of that of mimic NC controls (Figure 3a), suggesting that CXCR4 is a target of miR-9-5p during LSEC injury. The data were consolidated by western blot analysis, as the protein expression level of CXCR4 decreased by 51.8% following miR-9-5p overexpression (Figure 3b). Next, we determined whether changes in the expression pattern of miR-9-5p and CXCR4 impact on OGD/HG-induced LSEC injury. Indeed, our data showed that a 2.24-fold increase in miR-9-5p expression significantly improved the OD value of CCK-8 assay by 23.4 and 44.9% at 24 and 48 h post-injury, respectively (Figure 3c). In accordance with these findings, miR-9-5p overexpression also led to significant downregulation of pro-apoptotic proteins Bax, cleaved-caspase 3, and cleaved-caspase 9, as well as upregulation of anti-apoptotic protein Bcl-2 (Figure 3d). Furthermore, LSECs transfected with mimic miR-9-5p were also more resistant to inflammatory response, as determined by decrease expression levels of TNF-α, IL-1, and IL-6. In conclusion, our data indicate that miR-9-5p targets CXCR4 and exerts a protective role in OGD/HG-induced LSEC damage *in vitro* (Figure 4).

## 4 Discussion

Although tremendous effort has been made in the past decades to decipher the underlying mechanisms of I/R injury, therapies that successfully translated into clinical practice remain scarce [1,2]. The present study depicts a previously unidentified role of miR-9-5p in the LSEC pathological processes using an *in vitro* I/R model. We showed that miR-9-5p is downregulated and CXCR4 upregulated in an OGD/HG model in hepatic sinusoidal endothelial cells, suggesting their potential role in I/R injury. Upon OGD/HG insults, LSECs underwent enhanced oxidative stress, apoptosis, and inflammatory response, as well as aggravated cell death. Importantly, we revealed that CXCR4 is a downstream target of miR-9-5p. Indeed, overexpression of miR-9-5p diminished both mRNA and protein expression levels of CXCR4, thus preserving LSEC survival and suppressed the activation of Bax/Bcl-2 pro-apoptotic signaling pathway and synthesis of pro-inflammatory factors including TNF-α, IL-1, and IL-6 (Figure 4). Therefore, miR-9-5p/CXCR4 may represent a promising therapeutic target for LSEC protection during liver I/R injury.

Major liver surgery, including hepatectomy and transplantation, is the most effective and often the only treatment for liver cancer and other end-stage liver diseases [19,20]. Hepatic ischemia injury, initially because of reduced blood supply, is followed by reperfusion injury mediated by a variety of pathophysiological processes, a major complication referred to as liver I/R injury. This may be attributable to, at least in part, the therapeutic target cell type. The present study focuses on the LSECs. LSECs line the vascular endothelium of the hepatic tissue, which control vascular tone and thereby blood flow and delivery of nutrients and oxygen to hepatocytes. Thus, LSECs exhibit crucial biological functions including elimination of macromolecules and small particles from the blood supply, as well as immunological functions and facilitation of liver regeneration [21].

To the best of our knowledge, studies regarding the role of miR-9-5p in I/R injury is rudimentary, apart from our previous findings that it is vital for HUVEC survival during I/R injury. More importantly, given the essential role of LSECs in the maintenance of liver functioning, insight into the biological role of miR-9-5p in LSCEs is worthwhile. The conventional mouse model of liver I/R injury involves clamping the portal vein, bile duct, and hepatic artery with microvascular clips to interrupt the blood supply, followed by reperfusion [22,23]. The method, however, makes it difficult to elucidate cell type-specific role of the factor studied. Therefore, we used an *in vitro* model by incubating LSECs with OGD to mimic the low oxygen and glucose conditions as is experienced during liver transplantation. The LSECs were then treated with glucose-free DMEM medium for 6 h, followed by HG (30 mmol/L glucose) DMEM medium for 12 h. This enables us to observe the intracellular effects of I/R injury in LSCEs. We depict that miR-9-5p is fundamental for two pivotal pathological processes in LSECs, inflammatory response and apoptosis. However, further investigation is warranted to test the protective role of miR-9-5p *in vivo*.

Excessive inflammatory response is a key mechanism of liver IR injury. Previous studies indicate the involvement of various inflammatory cells and humoral factors in liver IR injury [5,6]. Upon I/R injury, activated SECs, neutrophils, and hepatocytes express pro-inflammatory cytokines and chemokines, such as TNF-α and IL-1, contributing to their damage. Previously, we showed that overexpression of miR-9-5p inhibited the release of inflammatory factors in HUVECs [18]. In contrast, a miR-9-5p inhibitor largely counteracted the anti-inflammatory effects upon CXCR4 inhibition in HUVEC [18], suggesting that miR-9-5p exerts anti-inflammatory effects. Nevertheless, whether miR-9-5p directly modulates this critical process in key hepatic components during I/R injury is less understood. Thus, we established an *in vitro* I/R injury model to test this hypothesis using LSECs. Our data demonstrates that TNF-α, IL-1, and IL-6 were all significantly downregulated in response to miR-9-5p overexpression in LSECs. In addition, IL-1 and IL-6 are also important mediators of ROS production [24,25,26,27]. Indeed, the enhanced production of TNF-α, IL-1, and IL-6 following OGD/HG insults is accompanied by increased OD value of MDA and decreased activity of GSH-PX, suggesting aggravated oxidative stress. In accordance with these data, we have shown previously that HUVECs overexpressing miR-9-5p exhibited opposite effects, i.e., decreased OD value of MDA and increased GSH-PX activity [18]. In line with our findings, recent studies showed an anti-inflammatory role of miR-9-5p in deep vein thrombosis and multiple sclerosis by targeting NF-κB [28,29,30]. Thus, the present study extended our knowledge of the potential targets of miR-9-5p during inflammatory response.

Apoptosis of liver-resident cells, a crucial mechanism of liver injury, is common under pathological condition of stress and diseases [31,32]. Given the small proportion of LSECs in the hepatic tissue, the importance of LSEC apoptosis, to some extent, may be underestimated. Intriguingly, miR-9-5p regulates apoptosis in various tissues and cell lines [13,33,34]. Its involvement in LSECs, however, remains unclear. Within minutes of I/R injury, LSECs undergo caspase-dependent apoptotic cell death, followed by that of hepatocyte death few hours later [31,32,35,36,37]. The present and our previous study showed that miR-9-5p-mediated anti-apoptotic effects may be beneficial for survival of various endothelial cell lines including HUVEC and LSECs [18]. Bax is also expressed in LSECs [4]. When looking into the mechanism of apoptosis reduction associated with miR-9-5p, we observed that overexpression of miR-9-5p elevated the protein expression levels of pro-apoptotic Bax, caspase-3, and caspase-9 and decreased the expression levels of anti-apoptotic Bcl-2, suggesting that miR-9-5p is a regulator of LSEC apoptosis following OGD/HG damage. Notably, an anti-apoptotic effect in LSECs may also prevent LSEC and hepatocyte injury [38]. Moreover, Tanoi et al. showed that inhibition of Bax in LSECs reduces apoptosis in both LSECs and hepatocytes [39]. In contrast to our finding, recent studies show that miR-9-5p may induce apoptosis in papillary thyroid cancer and oral squamous cell carcinoma [14,15]. This may be attributable to cell type, pathological conditions, and, more importantly, targeting factors.

microRNAs, approximately over 2,000 in human genome, are a class of small non-coding RNAs that inhibit gene expression [10,11]. They bind to complementary seed sequences in 3′-UTRs of mRNA, resulting in degradation or inhibition of the translation of target functional proteins [12]. Although the cause of reduction of miR-9-5p in response to I/R injury remains unknown, we have shown that OGD/HG insults sufficiently reduced the expression levels of miR-9-5p in LSECs. The binding sites of miR-9-5p and CXCR4 were predicted according to TargetScan (http://www.targetscan.org/vert_71/). However, whether such binding exerts a functional role needs to be tested. CXCR4 is a crucial mediator of hepatic, cardiac, and lung ischemia-reperfusion injury [40,41,42]. In the present study, we observed that the reduction of miR-9-5p correlated with enhanced expression of CXCR4 mRNA and protein levels. Overexpression of miR-9-5p significantly diminished CXCR4 expression, suggesting that CXCR4 is a target of miR-9-5p. Moreover, overexpression of miR-9-5p attenuated stress-induced apoptosis in LSEC. In accordance with these findings, our previous study showed that silencing of CXCR4 largely abrogated the pro-apoptotic and pro-inflammatory effects of miR-9-5p inhibition in HUVECs following HG insults [18]. In line with our findings, Lu et al. and Ferrer-Marin et al. reported that miR-9 strongly reduced CXCR4 expression in nasopharyngeal carcinoma and human megakaryocytes [16,17]. In addition, miR-9 directly binds to the 3′-UTRs of CXCR4 [16]. The precise role of miR-9-5p may depend on specific cell context. Regarding endothelial cells, the present and our previous study show that miR-9-5p targets CXCR4 and therefore attenuates stress-induced apoptosis in HUVEC and LSEC. It is possible that miR-9-5p/CXCR4 axis may play a broader role in pathological conditions such as cardiovascular disease and neurological diseases, where endothelial cells are also vital for the initiation and progression of the disease [18,43,44].

## 5 Conclusions

Our data indicate a pro-survival role of miR-9-5p in LSEC. Upon OGD/HG injury *in vitro*, the expression levels of miR-9-5p are downregulated, resulting in enhanced CXCR4 expression. Consequently, LSECs underwent aggravated apoptosis, excessive inflammatory response, and impaired cell survival. Taken together, miR-9-5p may be a promising therapeutic target to rescue endothelial cells during liver I/R injury.
